# High-entropy alloy catalysts with tunable electronic configurations for enhanced sulfur reduction electrocatalysis†

**DOI:** 10.1039/d5sc04586j

**Published:** 2025-07-21

**Authors:** Jingge Shi, Xu He, Hao Zhang, Wei Jiang, Ruizheng Zhao, Manman Wu, Yongzheng Fang, Menggai Jiao, Yiyang Liu, Zhen Zhou

**Affiliations:** a Interdisciplinary Research Center for Sustainable Energy Science and Engineering (IRC4SE^2^), School of Chemical Engineering, Zhengzhou University Zhengzhou Henan 450001 China liuyiyang@zzu.edu.cn zhenzhou@zzu.edu.cn; b Zhengzhou BAK Battery Co., Ltd Zhengzhou Henan 451450 China; c Longmen Laboratory Luoyang 471023 Henan China; d School of Materials Science and Engineering, Institute of New Energy Material Chemistry, Renewable Energy Conversion and Storage Center, Nankai University Tianjin 300350 China

## Abstract

The shuttle effect and sluggish redox kinetics of polysulfides pose significant challenges to the long-cycle stability of alkali metal–sulfur batteries, necessitating the development of highly efficient catalysts. High-entropy alloys (HEAs) have emerged as promising electrocatalysts for energy storage due to their unique electronic properties and high configurational entropy. Tailoring the electronic configuration of HEAs to achieve a well-positioned d-band center is a vital strategy for enhancing catalytic performance in alkali metal–sulfur batteries systems. In this study, the electronic configurations of HEAs were systematically tuned by varying the fifth metal element. Among them, NiCoFeCuMo (HEA-Mo) exhibited an optimized electronic configuration and a favorable d-band center, fully demonstrating the “cocktail effect” and thereby enhancing interactions with polysulfides. To evaluate its practical performance, HEA-Mo was integrated into polypropylene (PP) separators (HEA-Mo@PP) for Li–S and room-temperature Na–S batteries, both exhibiting excellent cyclic stability attributed to enhanced polysulfides adsorption and catalytic conversion. This work provides critical insight into the rational design of non-noble HEAs *via* electronic configuration modulation, offering a generalizable strategy for advancing next-generation energy storage systems.

## Abstract 2

Instant synthesis of the HEAs was achieved with optimized electronic configurations for ultra-stable, long cycle life alkali metal–sulfur batteries.

## Graphical abstract

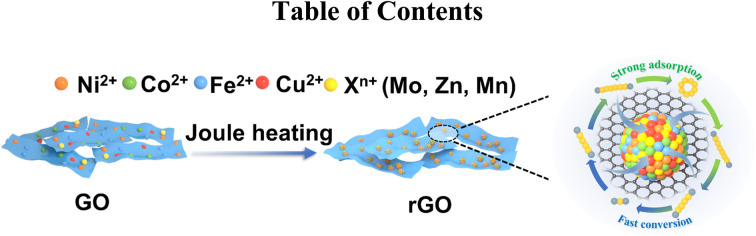

## Introduction

The rapid advancement of electric vehicles, drones, and energy storage power stations has created an urgent demand for high-performance energy storage devices that offer both long cycle life and high energy density.^1,2^ Among the promising candidates for next-generation energy storage are alkali metal–sulfur batteries, such as lithium–sulfur (Li–S) and room-temperature sodium–sulfur (Na–S) batteries, due to their high theoretical specific capacity (1675 mAh g^−1^), natural abundance, and environmental compatibility.^3,4^ However, several critical challenges hinder their practical application, including the intrinsic insulating nature of sulfur (S_8_) and short-chain discharge products, the shuttle effect caused by soluble long-chain polysulfides, significant volume changes during charge/discharge cycles, and sluggish redox kinetics of polysulfides conversion.^5,6^ To achieve high-performance, long-cycle alkali metal–sulfur batteries suitable for commercial deployment, the development of novel catalysts that can effectively accelerate sulfur redox reactions is urgently needed.

Extensive efforts have been dedicated to developing advanced materials such as porous carbon matrices,^7,8^ polar metal compounds,^9,10^ conductive polymers,^11^ heterostructures,^12–14^ and single-atom catalysts (SACs) to physically confine or chemically adsorb polysulfides. Though these strategies have led to notable progress, designing high-performance catalysts for sulfur reduction still faces fundamental challenges. Specifically, weak electrostatic interactions are often inadequate to suppress the pronounced shuttle effect of soluble polysulfides, whereas excessively strong chemisorption can hinder the subsequent redox reactions by trapping intermediates. Therefore, an effective catalytic system must simultaneously fulfill two critical criteria: strong and thermodynamically favorable immobilization of polysulfides to prevent diffusion, and fast redox kinetics to promote efficient sulfur conversion and ensure long-term cycling stability.^15,16^

Since its introduction in 2004,^17^ the concept of “high entropy” has garnered significant attention across various research disciplines. High-entropy alloys (HEAs), typically composed of five or more metallic elements forming a single-phase solid solution, have demonstrated considerable promise as efficient electrocatalysts in a range of reactions, including the oxygen reduction reaction (ORR),^18,19^ nitrogen reduction reaction (NRR),^20^ carbon dioxide reduction reaction (CO_2_RR),^21^ and sulfur reduction reaction (SRR).^22,23^ In alkali metal–sulfur batteries, the random distribution and interactions among multiple metallic elements in HEAs facilitate electron redistribution, thereby enhancing reaction kinetics. The synergistic “cocktail effect” of diverse metal components not only increases the density of active sites but also boosts their intrinsic catalytic activity, enabling strong polysulfide adsorption and efficient conversion.^24^ Additionally, lattice distortion in HEAs can optimize the electronic structure and engineer active sites, further enhancing catalytic performance.^25,26^ Owing to their unique multi-element synergy and tunable electronic configurations, HEAs are emerging as ideal candidates for achieving superior electrochemical performance in alkali metal–sulfur batteries.

HEAs exhibit tremendous potential in the field of electrocatalysis due to their unique multi-component structure and synergistic effects. To further highlight their advantages, comparative analyses between HEAs and other typical catalysts are presented herein. Taking single-atom catalysts (SACs) as an example, SACs have attracted extensive attention in alkali metal–sulfur batteries owing to their extremely high atomic utilization efficiency and well-defined active sites. However, SACs often suffer from inherent limitations, such as poor structural stability, tendency for metal atom aggregation, and a limited variety of catalytic sites, which may restrict their long-term electrochemical performance.^27,28^ In contrast, the intrinsic lattice distortion, cocktail effect, and multi-metal synergistic interactions of HEAs not only endow them with excellent thermal and chemical stability but also result in abundant active sites with a wide spatial distribution and tunable electronic structures. Furthermore, unlike SACs, which possess isolated and singular reaction sites, HEAs provide multi-component catalytic centers that facilitate the synergistic catalysis of multi-step reaction pathways, thereby promoting faster redox kinetics. These advantages make HEAs an outstanding electrocatalyst, particularly suitable for complex multi-step reactions involved in polysulfide conversion within alkali metal–sulfur batteries.

In this study, three non-noble HEA catalysts NiCoFeCuX (denoted as HEA-X, where X = Mo, Zn, Mn), were rapidly synthesized *via* a simple Joule-heating treatment for separator modification in alkali metal–sulfur batteries. By combining four common transition metals and tuning the fifth component based on outer electron arrangement, the electronic configurations of the HEAs were effectively modulated. Density functional theory (DFT) calculations revealed that HEA-Mo possesses the most advantageous d-band center and strongest polysulfides adsorption capability, indicating its optimized electronic configuration and superior catalytic potential. DFT analyses further reveal that HEA-Mo effectively lowers energy barriers associated with liquid–solid and solid–solid phase transitions, thereby accelerating sulfur reduction reaction, suppressing polysulfides shuttling. Correspondingly, Li–S batteries assembled with the HEA-Mo@PP exhibit remarkable practical performance, with a low-capacity decay rate of only 0.027% per cycle over 2000 cycles at 1C. Furthermore, the HEA-Mo@PP was further applied to room temperature Na–S batteries, demonstrating a decay rate of only 0.018% per cycle after 2000 cycles at 2C. These results not only highlight the effectiveness of electronic configuration tuning in designing high-performance HEA catalysts but also pave the way for their broad application in next-generation energy storage systems.

## Results and discussion

### Preparation and characterization of HEAs

**Fig. 1:**
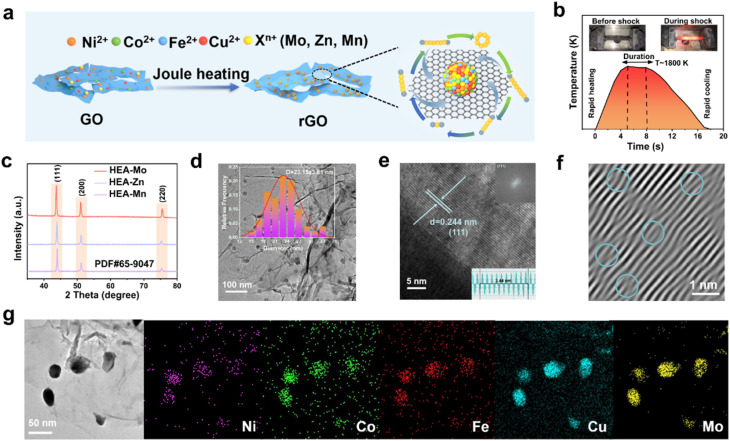
Preparation and structural characterizations of HEA-X. (a) Schematic illustration of the preparation of HEA-X and the (b) the ultrafast calcination process. (c) XRD patterns; (d) TEM images; the inset shows the calculated particle size distribution. (e) HRTEM image with corresponding IFFT patterns, (f) lattice distortions, and (g) HAADF-STEM images with EDS elemental mapping.

Four common transition metal ions (Ni(ii), Co(ii), Fe(ii), and Cu(ii)) were selected as metal sources, while a fifth metal ion (X, where X = Mo(v), Zn(ii), or Mn(ii)) was varied across samples. All five metal ions were co-dissolved in an ethanol solution containing dispersed graphene oxide (GO). After drying, GO underwent thermal reduction *via* the ultra-high-temperature Joule heating process, forming reduced GO (rGO) (Fig. S1†). Simultaneously, the metal ions were reduced to atoms, forming numerous disordered molten droplets, which subsequently coalesced into HEA nanoparticles that uniformly adhered to rGO during rapid annealing (Fig. 1a and b).

X-ray diffraction (XRD, Fig. 1c) patterns confirm the successful preparation of HEAs (PDF#65-9047) with a face-centered cubic (FCC) crystal structure. The diffraction peaks correspond to the (111), (200), and (220) crystal planes of the HEAs at 43.9°, 51.2°, and 75.4°, respectively. Notably, the (111) peak positions exhibit varying degrees of leftward shifts relative to the standard reference, likely due to the difference in the atomic sizes of Mo, Zn, and Mn (Fig. S2†).

Additionally, HEA-Mo displays significantly higher peak intensities compared with HEA-Zn and HEA-Mn, indicating superior crystallinity. Transmission electron microscope (TEM) images reveal the uniform attachment of HEA nanoparticles to rGO, with an average particle size of ∼23 nm (Fig. 1d, S3 and S4†). High-resolution TEM (HRTEM) and fast inverse Fourier transformation (IFFT) further determine the lattice fringe spacings of HEA-Mo, HEA-Zn, and HEA-Mn as 0.244 nm, 0.243 nm, and 0.243 nm, respectively (Fig. 1e, S3 and S4†), corresponding to the (111) crystal plane of HEAs. These differences in lattice stripes correlate with the observed peak shifts in the XRD analysis.

The lattice distortion in HEAs (Fig. 1f and S5(b)†) plays a crucial role in enhancing catalytic activity by modulating electronic structure, optimizing adsorption energy, and stabilizing defect sites.^29^ Furthermore, energy dispersive spectroscopy (EDS) (Fig. 1g, S3 and S4†) confirms the homogeneous distribution of the five elements. As summarized in Table S1 (see details in ESI),† the entropy values of HEAs satisfy the criteria for high-entropy materials (*S* ≥ 1.5 R). The inherent conformational entropy stabilization effect of HEAs grants them exceptional properties that surpass conventional alloys, making them ideal candidates for advanced catalytic materials in alkali metal–sulfur batteries.

### Polysulfide adsorption

The HEA-modified separators (HEA-X@PP) were prepared by mixing HEA-X with polyvinylidene fluoride (PVDF) to form a slurry, which was then blade-coated onto a polypropylene (PP) separator with a coating thickness of ∼10 μm (Fig. S6†). The coated side was oriented toward the S cathode to effectively suppress the shuttle effect of lithium polysulfides (LiPSs), thereby enhancing sulfur utilization in Li–S batteries.

The adsorption capacities of PP and HEA-X@PP interlayers were evaluated through Li_2_S_6_ permeation experiments (Fig. S7†). Visual observations revealed distinct LiPS diffusion behaviors: after 24 hours, the HEA-Mo@PP interlayer maintained a colorless solution, while HEA-Zn@PP and HEA-Mn@PP interlayers turned light yellow. In contrast, the PP control group exhibited rapid Li_2_S_6_ diffusion, resulting in a dark yellow solution. This stark difference highlights the superior polysulfide-trapping capability of HEA-Mo@PP.

**Fig. 2:**
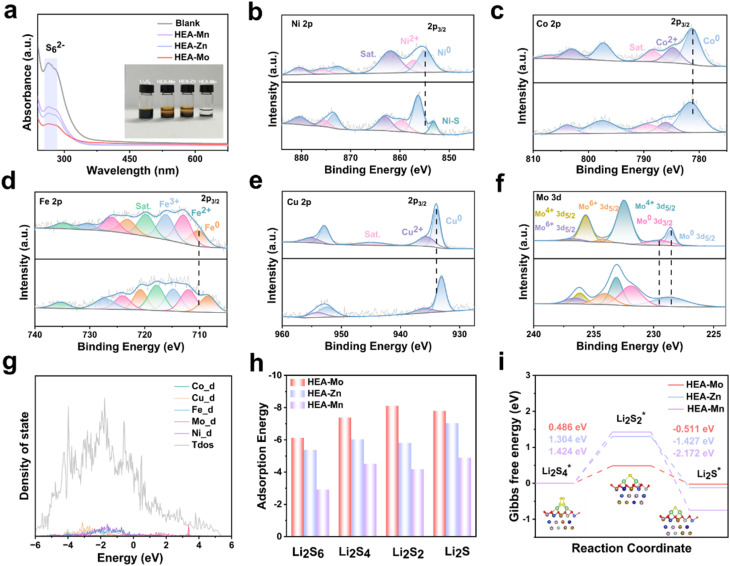
Adsorption performance of HEA-X. (a) UV-vis absorption spectra of a Li_2_S_6_ solution before and after the addition of HEA-Mo, HEA-Zn, and HEA-Mn, and the inset shows a photograph of a Li_2_S_6_ visual adsorption after 24 h. (b–f) High resolution XPS of Ni 2p, Co 2p, Fe 2p, Cu 2p, and Mo 3d of HEA-Mo before and after adsorption of Li_2_S_6_. (g) Total density of states of HEA-Mo. (h) Adsorption energies of LiPSs on HEAs surfaces. (i) Gibbs free energy profile of Li_2_S_4_ → Li_2_S_2_ → Li_2_S on HEAs.

To evaluate the effectiveness of HEAs in inhibiting LiPSs shuttling, Li_2_S_6_ adsorption tests were conducted. After 24 hours, the solution containing HEA-Mo remained nearly transparent, whereas the addition of HEA-Zn and HEA-Mn resulted in a light-yellow coloration compared with the Li_2_S_6_ solution. These results indicate that HEA-Mo exhibits the strongest adsorption capability for Li_2_S_6_ (Fig. 2a). Ultraviolet-visible (UV-vis) absorption spectroscopy further supports this finding, as the solution containing HEA-Mo displayed the lowest characteristic peak intensity within the 250–300 nm range, consistent with the adsorption tests.

To investigate the chemical interactions between HEAs and Li_2_S_6_, XPS of HEA-Mo before and after adsorption were compared (Fig. 2b–f). Taking Ni 2p_3/2_ as an example, the broad peaks, consisting of two components at 854.91 and 857.18 eV, correspond to Ni^0^ and Ni^2+^, respectively. After Li_2_S_6_ adsorption, these peaks shift to 856.30 and 859.69 eV due to electron transfer from Ni atoms to Li_2_S_6_. Additionally, the emergence of a new peak at 853.13 eV, attributed to Ni–S interaction, confirms the formation of Ni–S bonding.

Interestingly, significant shifts toward higher binding energies were also observed in the XPS of Co 2p and Mo 3d after adsorption, whereas Fe 2p and Cu 2p exhibited shifts toward lower binding energies. This trend was similarly observed in HEA-Zn (Fig. S8†) and HEA-Mn (Fig. S9†). The observed shifts in XPS binding energies following Li_2_S_6_ adsorption suggest a complex electron transfer within the alloy. Given the strong electronegativity of S atoms, electrons are withdrawn from Ni, Co, and Mo, reducing their electron cloud density and increasing binding energy. To maintain overall charge balance, electrons are redistributed from Li_2_S_6_ and other alloy regions toward Fe and Cu atoms, leading to increased electron density around Fe and Cu and consequently lowering their binding energies.

This electron transfer-compensation mechanism is an inherent self-regulatory behavior of HEAs, allowing the system to reach a new energy equilibrium during adsorption. This dynamic charge redistribution is a unique characteristic of HEA-based catalysts, enabling precise regulation of charge distribution and optimization of adsorption energies, thereby enhancing catalytic performance.

To explore the catalytic effect of the three HEAs on sulfur reduction at an atomic scale, density functional theory (DFT) calculations were conducted, after Monte Carlo (MC) simulations were first employed to determine the equilibrium atomic configurations of metal atoms in the HEA models (Fig. S10†), establishing stable structures for further analysis. During HEAs synthesis, metal nitrates were used as precursors. Due to incomplete reduction, residual oxygen species were likely introduced on the surface. To accurately represent the actual chemical state, simulations employed oxygen-saturated HEA surface structures. According to d-band center theory, a closer proximity to the Fermi level generally enhances LiPSs adsorption capacity.^30^ The d-band center of HEA-Mo (−0.535 eV) was found to be closer to the Fermi level compared with HEA-Zn (−0.795 eV) and HEA-Mn (−1.166 eV) (Fig. 2g and S11†), indicating that HEA-Mo possesses a more advantageous electronic configuration. This optimized electronic configuration facilitates enhanced LiPSs adsorption and catalytic conversion, underpinning its superior catalytic performance.

Subsequently, adsorption energies for LiPSs on the optimized (111) crystal plane of HEA-X were calculated (Fig. 2h and S12–S14†). The DFT results revealed that HEA-Mo exhibited superior LiPSs adsorption performance, with an adsorption energy of −6.12 eV for Li_2_S_6_, significantly higher than HEA-Zn (−5.37 eV) and HEA-Mn (−2.91 eV) by 0.75 eV and 3.21 eV, respectively. This trend remained consistent for other sulfides, underscoring HEA-Mo's superior LiPS immobilization capability. Taking the key intermediate of the liquid–solid reaction, Li_2_S_4_, as an example, we investigated the charge redistribution behavior between lithium polysulfides and three HEA catalysts by performing differential charge density analysis (Fig. S15†). As shown in Fig. S15,† there is significant charge transfer between the three high-entropy alloys and lithium polysulfide, thereby effectively activating the lithium polysulfide. We further conducted projected crystal orbital Hamilton population (pCOHP) calculations to gain deeper understanding of the bonding–antibonding properties of the S–S bond in Li_2_S_4_ in the surface of the three HEAs.^31^ As depicted in Fig. S16,† HEA-Mo exhibits the lowest occupancy of bonding states in the S–S bond among the three catalysts, indicating its superior ability to activate Li_2_S_4_, which significantly reduce the energy barrier for S–S bond cleavage. This finding is further supported by the calculated integrated COHP (ICOHP) values below the Fermi level, which are −4.58, −4.62, and −4.82 eV for HEA-Mo, HEA-Zn, and HEA-Mn, respectively. Overall, the alteration in electronic properties of HEAs significantly impact their adsorption and activation capability for lithium polysulfide, thereby altering the reaction kinetics.

To further investigate LiPS conversion, Gibbs free energy calculations were conducted to analyze the conversion steps. Since the liquid–solid (Li_2_S_4_ to Li_2_S_2_) and solid–solid (Li_2_S_2_ to Li_2_S) transformations are recognized as the rate-determining steps in sulfur reduction processes,^32^ their associated energy barriers serve as key indicators of catalytic performance. As shown in Fig. 2i, the energy barrier for the Li_2_S_4_ to Li_2_S_2_ conversion is 0.486 eV for HEA-Mo, significantly lower than the barriers of 1.304 eV and 1.424 eV for HEA-Zn and HEA-Mn, respectively. This suggests that HEA-Mo is highly effective in promoting LiPS conversion. These calculations provide valuable insights that can guide the screening and optimization of sulfur reduction catalysts.

### Kinetic analyses and catalytic performance

**Fig. 3:**
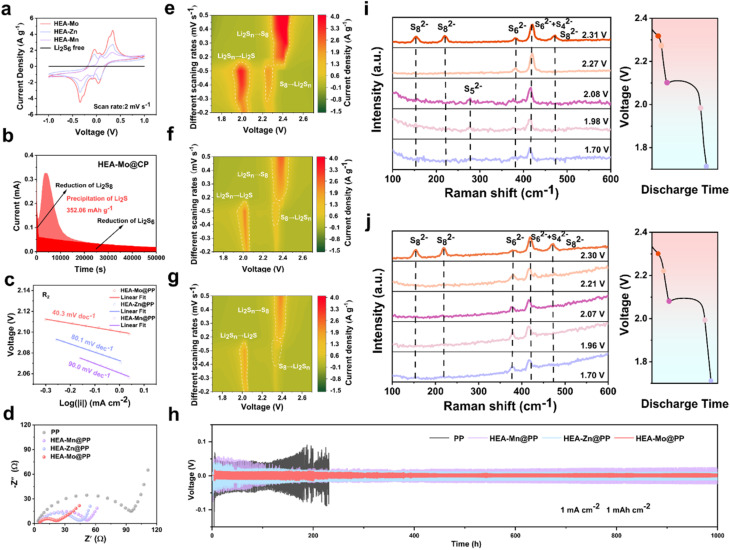
Interaction with LiPSs and accelerated reaction kinetics. (a) CV curves of Li_2_S_6_ symmetrical cells with HEA-X and without catalyst at the scan rate of 2 mV s^−1^. (b) Li_2_S deposition measurements HEA-Mo, with potentiostatic discharge profile of 2.05 V. (c) Tafel plots derived from CV curves at 0.2 mV s^−1^ calculated from the reduction peaks R_2_. (d) Nyquist plots for EIS profiles of Li–S batteries with different separators. Contour maps of CV curves for (e) HEA-Mo, (f) HEA-Zn, and (g) HEA-Mn at various scan rates (0.2–0.5 mV s^−1^). (h) Voltage profiles of symmetric cells with PP, HEA-Mn@PP, HEA-Zn@PP, and HEA-Mo@PP separators, tested at 1 mA cm^−2^ and 1 mAh cm^−2^. *In situ* Raman spectra and corresponding discharge curves of the HEA-Mo modified separator (i) and PP (j) at different voltages.

The catalytic activity of the HEA catalysts is assessed based on the overall oxidation–reduction processes in Li–S batteries, effectively evaluated *via* CV. To experimentally examine the electrocatalytic activity of the designed interlayers in LiPSs conversion, symmetric cells with identical HEA-X electrodes were constructed with an electrolyte containing Li_2_S_6_, followed by CV testing between −1.0 V and 1.0 V at a scan rate of 2 mV s^−1^.^33^ The CV curves of symmetric cells (Fig. 3a) clearly demonstrate that the HEA-Mo electrode exhibits higher peak currents and distinct redox peaks, indicating enhanced redox kinetics between Li_2_S and S_8_. These results collectively confirm the superior catalytic activity of HEA-Mo to HEA-Zn and HEA-Mn.

To verify the catalytic role of HEAs in Li_2_S deposition, kinetic analyses of the liquid–solid conversion were conducted. The results reveal that the HEA-Mo@CP electrode facilitated the highest Li_2_S deposition of 352 mAh g^−1^, significantly surpassing HEA-Zn@CP (143 mAh g^−1^) and HEA-Mn@CP (75.4 mAh g^−1^) (Fig. 3b and S17†). These findings highlight HEA-Mo's remarkable ability to enhance Li_2_S nucleation, underscoring its superior catalytic function.

Additionally, variable sweep rate CV curves (Fig. S18†) were recorded within the range of 1.7–2.7 V *vs.* Li/Li^+^ with different modified separators. Peak R_1_ represents the conversion of S_8_ to soluble LiPSs, while peak R_2_ indicates the transition from soluble LiPSs to Li_2_S, both representing key steps in the sulfur reduction process. Peak O_1_ corresponds to Li_2_S oxidation back to S_8_.

The linear relationship between the peak currents (*I*) of R_2_ and O_1_ with the square root of the scan rate (*ν*^0.5^) enabled calculation of Li^+^ diffusion coefficients for the symmetric cells with modified separators with the Randles–Sevcik equation (Fig. S19†). The results indicate that HEA-Mo-modified separators exhibit faster Li^+^ diffusion and improved redox kinetics compared with HEA-Zn and HEA-Mn.

The Tafel plot derived from the peak R_2_ of the CV curve at a scan rate of 0.2 mV s^−1^ (Fig. 3c) reveals that the fitted slope value of HEA-Mo (40.3 mV dec^−1^) is lower than that of HEA-Zn (80.1 mV dec^−1^) and HEA-Mn (90.0 mV dec^−1^). Similarly, the Tafel slope values for peak O_1_ (Fig. S20†) also demonstrate that HEA-Mo (79.7 mV dec^−1^) has lower values than HEA-Zn (95.3 mV dec^−1^) and HEA-Mn (109.1 mV dec^−1^). The smaller Tafel slope of HEA-Mo suggests an accelerated kinetic rate of LiPS conversion, thereby facilitating the reduction process of S_8_ and the oxidation process of Li_2_S.

EIS analyses show that the HEA-Mo@PP-based Li–S batteries exhibit lower impedance in the high-frequency region (Fig. 3d), indicating accelerated charge transfer kinetics at the electrode–electrolyte interface. Furthermore, contour plots of the CV curves at different scanning speeds (0.2–0.5 mV s^−1^) are presented in Fig. 3e–g. Comparative analyses with HEA-Zn and HEA-Mn reveal that HEA-Mo exhibits higher currents, indicative of faster reaction kinetics. Overall, the HEA-Mo catalyst enhanced the overall sulfur conversion reaction kinetics and promoted rapid charge transfer, both of which are crucial for optimizing the performance of Li–S batteries.

To further understand the conversion process of LiPSs and to investigate the inhibition of polysulfide shuttling behavior by HEA-Mo, the discharge process of Li–S cells with HEA-Mo@PP and bare PP were monitored with *in situ* Raman spectroscopy (Fig. 3i and j). During discharge, specific Raman signals corresponding to S_8_^2−^ (153.3, 219.5, and 472.5 cm^−1^), S_4_^2−^ + S_6_^2−^ (419.8 cm^−1^), and S_6_^2−^ (381.0 cm^−1^) were detected at 2.31 V. As the discharge continued to 2.27 V, the intensity of the S_8_^2−^ peak disappeared completely, and the signal for S_6_^2−^ weakened. Subsequently, at 2.08 V, the signal peak for S_5_^2−^ (277.7 cm^−1^) appeared. As the discharge process continues, both S_6_^2−^ and S_5_^2−^ peaks disappeared, leaving only a signal peak of 419.8 cm^−1^ at 1.7 V. HEA-Mo was found to accelerate the conversion of polysulfides to lower-order materials during the discharge process, thereby reducing the formation and accumulation of higher-order polysulfides. In contrast to the HEA-Mo@PP, the bare PP showed the presence of S_6_^2−^ and S_4_^2−^ + S_6_^2−^ signals throughout the discharge process, indicating incomplete conversion, which was further manifested by the loss of active sulfur material and the degradation of cell performance.^34^

To evaluate lithium plating and stripping behavior, Li–Li symmetric cells were constructed with HEA-Mo@PP, HEA-Zn@PP, HEA-Mn@PP, and bare PP separators. The lithium plating/stripping performance of these cells was assessed under galvanostatic conditions at 1 mA cm^−2^ and 1 mAh cm^−2^ conditions (Fig. 3h). Notably, cells with bare PP separators failed abruptly after only 231 hours due to lithium dendrite growth. In contrast, the cells with HEA-Mo@PP demonstrated an impressive cycle life of 1000 hours, maintaining remarkably stable polarization at ∼34 mV. To further elucidate the underlying mechanism, post-cycling morphological analysis of the lithium metal anodes was conducted through SEM (Fig. S21†). The lithium surfaces in the symmetric cells with the HEA-Mo@PP modified separator are significantly smoother and more compact than those with HEA-Zn@PP and HEA-Mn@PP, indicating a more uniform lithium nucleation and deposition process. Such uniformity effectively suppresses the formation of lithium dendrites during repeated plating/stripping cycles, thereby enhancing interfacial stability and contributing to the superior long-term cycling performance of the batteries.

### Electrochemical performance of Li–S batteries

**Fig. 4:**
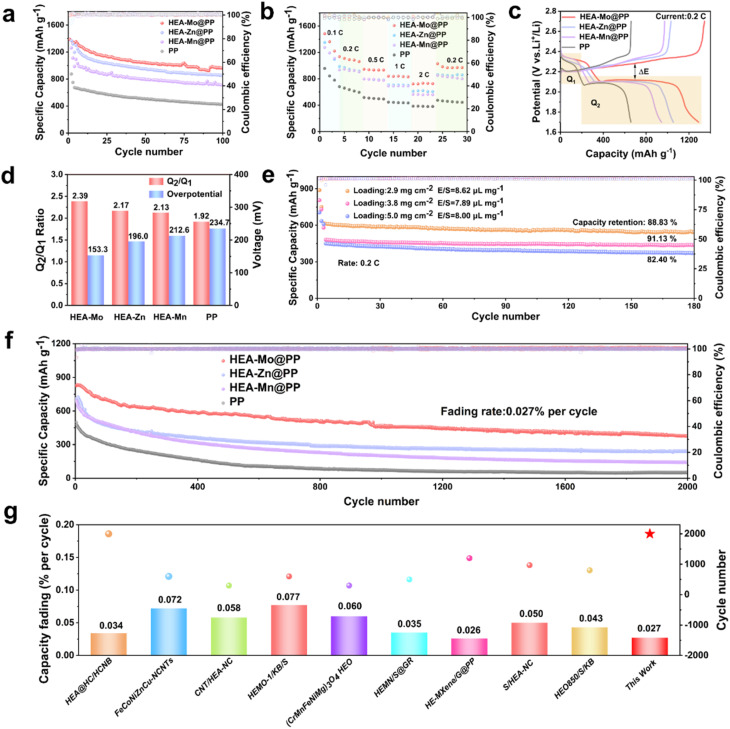
Electrochemical performance of Li–S batteries. (a) Cycling performance of cells with HEA-X-based modified separators and catalyst-free separator at 0.2C. (b) Rate capabilities. (c) GCD curves of different separators at a current rate of 0.2C. (d) Δ*E* and *Q*_2_/*Q*_1_ values. (e) Cycling performance of cells with HEA-Mo-based modified separators at different loadings. (f) Cycling performance of different separators at 1C. (g) Comparison of cycling performance with literature.

The impact of HEA-X@PP in Li–S batteries were evaluated in batteries with separators with different coatings. The cycling tests at 0.2C (1C = 1675 mAh g^−1^) revealed that batteries with HEA-Mo@PP separators exhibited a high initial discharge capacity (IDC) of 1337.4 mAh g^−1^, with a remaining specific capacity of 965.6 mAh g^−1^ after 100 cycles. This performance exceeded that of batteries with HEA-Zn@PP (859.8 mAh g^−1^), HEA-Mn@PP (727.0 mAh g^−1^) and bare PP (427.3 mAh g^−1^) interlayers (Fig. 4a). The rate capability of Li–S batteries with different separators was evaluated from 0.1 to 2C (Fig. 4b). The Li–S batteries equipped with HEA-Mo@PP separators delivered specific capacity of 1483, 1137.2, 945.4, 839.6, and 733.7 mAh g^−1^ at 0.1C, 0.2C, 0.5C, 1C, and 2C, respectively. These results significantly surpassed those achieved by Li–S batteries assembled with other separators.

In the galvanostatic charge–discharge (GCD) platform, *Q*_1_ represents the initial discharge stage, corresponding to the solid–liquid conversion of S_8_ to Li_2_S_4_, which involves a 4-electron reaction. *Q*_2_ represents the conversion of Li_2_S_4_ to the final product Li_2_S, a liquid–solid transition involving a 12-electron reaction, typically considered the rate-determining step (Fig. 4c). The discharge curves indicate that the *Q*_2_ stage accounts for the majority of the capacity, and the *Q*_2_/*Q*_1_ ratio serves as a metric for assessing the catalytic activity of LiPS conversion.^35^ Additionally, voltage polarization (Δ*E* = *E*_charge_ − *E*_discharge_) between charging and discharging curves was analyzed to elucidate the sulfur conversion process. The *Q*_2_/*Q*_1_ ratio for HEA-Mo@PP (2.39) was significantly higher than that of HEA-Zn@PP (2.17), HEA-Mn@PP (2.13), and PP (1.92), highlighting HEA-Mo's superior catalytic activity in liquid-to-solid conversion (Fig. 4d).

Furthermore, the Δ*E* value of HEA-Mo@PP (153.3 mV) was notably lower than those of HEA-Zn@PP (196.0 mV), HEA-Mn@PP (212.6 mV), and PP (234.7 mV), indicating that HEA-Mo effectively accelerates the reversible redox process. The HEA-Mo@PP-based cells exhibited the lowest polarization for Li_2_S activation during charging, underscoring their advantage in bidirectional catalysis.

The long-term cycling performance of HEA-X@PP in Li–S batteries was evaluated at 1C to confirm its application potential. Cycling tests (Fig. 4f) demonstrate that HEA-Mo@PP exhibited remarkable stability over 2000 cycles, with a capacity decay rate of only 0.027% per cycle, significantly lower than HEA-Zn@PP (0.033%) and HEA-Mn@PP (0.039%). In contrast, the bare PP interlayer showed rapid degradation, with its IDC dropping from 498.2 mAh g^−1^ to 51.5 mAh g^−1^ (capacity decay: 0.045% per cycle). This striking contrast highlights HEA-Mo's catalytic ability in facilitating the bidirectional reaction of LiPSs, ultimately enhancing LiPS conversion efficiency.

Comparative analysis with previously reported HEAs (Fig. 4g),^36–44^ and several recently developed representative sulfur electrocatalysts (Table S2, see details in ESI†), including heterostructures, metal phosphides, and single-atom catalysts, further underscores HEA-Mo exhibits highly competitive electrochemical performance in Li–S batteries. Notably, HEA-Mo demonstrates a significantly lower capacity decay rate than many state-of-the-art catalysts. This superior performance is primarily attributed to the synergistic interactions among multiple transition metals within the HEAs, which enhance LiPS conversion kinetics and accelerate the overall sulfur redox processes. These results highlight the emerging design rationale for HEA-based catalysts: their compositional complexity enables tailored electronic structures, a high density of active sites, and excellent chemical stability, collectively making HEAs highly promising candidates for advanced Li–S batteries applications.

To further validate the catalytic superiority of HEA-Mo@PP, its performance was assessed in Li–S batteries with higher sulfur loading. After 180 cycles at 0.2C with sulfur loadings of 2.7 mg cm^−2^, 3.8 mg cm^−2^, and 5.0 mg cm^−2^, capacity retention rates were observed at 88.83%, 91.13%, and 82.40%, respectively (Fig. 4e). These results highlight HEA-Mo@PP's exceptional cycling stability even under high sulfur-loading conditions. The superior performance can be attributed to HEA-Mo@PP's ability to efficiently regulate LiPS conversion during operation at elevated sulfur loadings.

### Electrochemical performance of room-temperature Na–S batteries

**Fig. 5:**
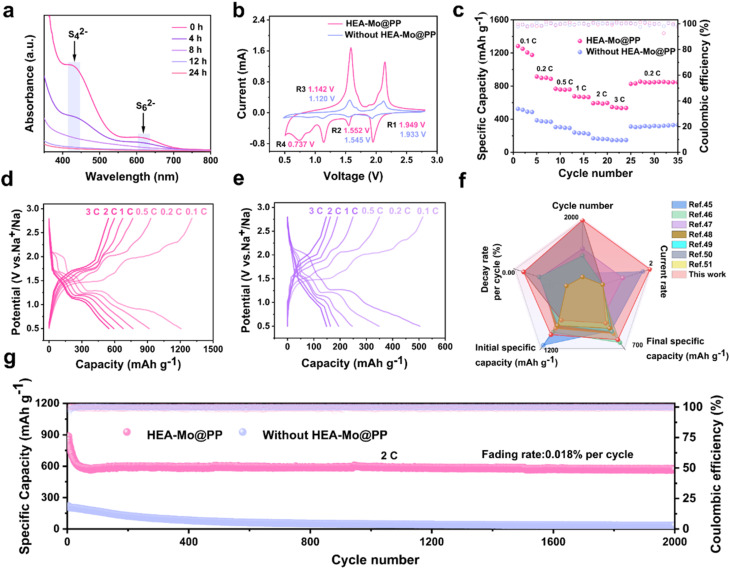
Electrochemical performance of room-temperature Na–S batteries. (a) UV-vis absorption spectra of Na_2_S_6_ solution after addition of HEA-Mo at different time intervals. (b) CV profiles of Na–S batteries with different separators. (c) Rate capability of different separators at specific currents rates of 0.1C, 0.2C, 0.5C, 1C, 2C, and 3C. Galvanostatic discharge–charge profiles at various current density with HEA-Mo-based modified separators (d) and catalyst-free separators (e). (f) Comparative radar chart of electrochemical properties. (g) Long-term cycling performance of HEA-Mo@PP and catalyst-free separators at 1C.

To further validate the bifunctional effectiveness of HEA-Mo in suppressing polysulfides shuttling and enhancing sulfur redox kinetics, HEA-Mo@PP was employed in room-temperature Na–S batteries. UV-vis spectroscopy was utilized to assess HEA-Mo's adsorption capability in a Na_2_S_6_ solution at various time intervals (Fig. 5a). The pristine Na_2_S_6_ solution exhibited characteristic absorption peaks at 426 nm and 617 nm, corresponding to S_4_^2−^ and the chain-like structure of S_6_^2−^, respectively. After 24 hours, the solution became nearly colorless (Fig. S22†). Notably, significant adsorption occurred within the initial hours, with pronounced effects observed at 4 and 8 hours. EIS revealed a lower interfacial resistance for HEA-Mo@PP compared with the unmodified separator (Fig. S23†). CV measurements further demonstrated that HEA-Mo@PP-based Na–S batteries exhibited higher peak current density and an earlier reduction potential, highlighting its superior catalytic activity and kinetic properties (Fig. 5b).^45^

Similarly, HEA-Mo@PP showed exceptional rate capability, delivering a specific capacity of 547.1 mAh g^−1^ at a high rate of 3C, whereas the separator without HEA-Mo achieved only 146.7 mAh g^−1^ (Fig. 5c). The capacity–voltage profiles (Fig. 5d and e) confirm that HEA-Mo@PP-based room-temperature Na–S cells exhibited a higher specific capacity than the bare separator.^46^ HEA-Mo@PP demonstrated an initial specific capacity of 1192.4 mAh g^−1^, retaining 986.5 mAh g^−1^ after 100 cycles at 0.2C (Fig. S24†). In contrast, the room-temperature Na–S batteries without HEA exhibited a substantially lower capacity of only 432.9 mAh g^−1^. Furthermore, HEA-Mo@PP delivered a high IDC of 885.4 mAh g^−1^ and maintained a reversible capacity of 575.5 mAh g^−1^ after 2000 cycles at 2C, exhibiting an ultralow decay rate of 0.018% per cycle. In stark contrast, the batteries with the separator without HEA-Mo retained only 34.6 mAh g^−1^ after cycling (Fig. 5g).

Despite its modest initial specific capacity, HEA-Mo@PP-based room-temperature Na–S batteries demonstrated remarkable cycling stability at 2C, exhibiting significantly superior cycle life and a lower capacity decay rate per cycle compared with state-of-the-art catalysts (Fig. 5f).^47–53^

Accordingly, HEA-Mo@PP exhibits exceptional polysulfides adsorption capability and superior catalytic activity for sulfur redox reactions in room-temperature Na–S batteries. These synergistic functionalities significantly enhance rate performance, cycling stability, and capacity retention, validating the bifunctional role of HEA-Mo in regulating polysulfide behavior and optimizing redox kinetics.

## Conclusion

In summary, the electronic configurations of HEAs were systematically tuned by varying the fifth metal element. Through a combination of computational modeling and experimental validation, HEA-Mo was identified as an optimal candidate due to its superior polysulfides adsorption capability and remarkable catalytic activity in promoting polysulfide conversion. This work not only underscores the potential of HEA-Mo for next-generation energy storage systems but also provides valuable insights into the rational design of HEA-based materials. Future research should focus on further optimizing HEA compositions and exploring their adaptability to broader energy applications, paving the way for the development of high-performance alkali metal–sulfur batteries with long-term cycling stability.

## Author contributions

J. Shi: investigation & resources, data analysis, writing – original draft; X. H.: calculation, data discussion; H. Zhang: data analysis; W. Jiang: data analysis; R. Zhao: conceptualization; M. Wu: conceptualization, data discussion; Y. Fang: experimental design, data discussion, revise the manuscript; M. Jiao: calculation, data discussion, revise the manuscript; Y. Liu: validation, data analysis, revise the manuscript, project administration; Z. Zhou: conceptualization, supervision, data analysis, revise and finalize the manuscript.

## Conflicts of interest

The authors declare no conflict of interest.

## Supplementary Material

SC-016-D5SC04586J-s001

## Data Availability

The original data supporting this article are available in the main text and ESI.†
