# Supplementary material for: High-entropy alloy catalysts with tunable electronic configurations for enhanced sulfur reduction electrocatalysis
Source: Chem Sci. 2025 Jul 21;16(33):14956–66. doi: 10.1039/d5sc04586j (PMC12301893; doi:10.1039/d5sc04586j)
Supplement: SC-016-D5SC04586J-s001 [file SC-016-D5SC04586J-s001.pdf]

Supplementary Information (SI) for Chemical Science.
This journal is © The Royal Society of Chemistry 2025

Electronic supplementary information (ESI)

High-Entropy Alloy Catalysts with Tunable Electronic Configurations for Enhanced Sulfur Reduction Electrocatalysis

Jingge Shi[a,#], Xu He[a,#], Hao Zhang[a], Wei Jiang[b], Ruizheng Zhao[a], Manman Wu[a], Yongzheng Fang[a,c], Menggai Jiao[a], Yiyang Liu[a,*], Zhen Zhou[a,d,*]

[a] Interdisciplinary Research Center for Sustainable Energy Science and Engineering (IRC4SE[2]), School of Chemical Engineering, Zhengzhou University, Zhengzhou, Henan 450001, China

[b] Zhengzhou BAK Battery Co., Ltd, Zhengzhou, Henan 451450, China

[c] Longmen Laboratory, Luoyang 471023, Henan, China

[d] School of Materials Science and Engineering, Institute of New Energy Material Chemistry, Renewable Energy Conversion and Storage Center, Nankai University, Tianjin 300350, China.

*Corresponding author: Yiyang Liu (liuyiyang@zzu.edu.cn); Zhen Zhou (zhenzhou@zzu.edu.cn)

Experimental Details

Materials and reagents

All chemicals and solvents in this work were obtained commercially and used without further purification. Ethanol absolute (EtOH, 99.9%) was purchased from Beijing InnoChem Science & Technology Co., Ltd. Super p and Ketjenblack (KB) was purchased from Shenzhen Kejingzhida Technology Co., Ltd. Graphene Oxide (GO) was purchased from Hangzhou Gaoxi Technology Co., Ltd. Nickel nitrate hexahydrate ($Ni(NO_3)_2·6H_2O$,99.99%), Copper nitrate trihydrate ($Cu(NO_3)_2·3H_2O$,99.99%), Cobalt nitrate hexahydrate ($Co(NO_3)_2·6H_2O$,99.99%), Ferric nitrate hydrate ($Fe(NO_3)_3·6H_2O$,99.99%), Manganese nitrate tetrahydrate ($Mn(NO_3)_2·4H_2O$,98.0%), Zinc nitrate hexahydrate ($Zn(NO_3)_2·6H_2O$,99.99%), Molybdenum chloride ($MoCl_5$,99.6%) and Sublimated sulfur (S, 100 mesh) were purchased from Shanghai Aladdin Biochemical Technology Co., Ltd. Poly(1,1-difluoroethylene) (PVDF) and electrolytes were provided by Suzhou Duoduo Chemical Technology Co., Ltd. 1,3-Dioxolane (DOL,99.5%) and 1,2-dimethoxyethane (DME,99.5%) were purchased from Shanghai Myriad Chemical Technology Co., Ltd.

Synthesis of HEA-X

Firstly, 100 mg of Graphene Oxide (GO) was weighed and dissolved in 100 ml of anhydrous ethanol, stirred for 30 min, as solution I. 29.1 mg (0.1 mmol) $Ni(NO_3)_2·6H_2O$, 29.1 mg (0.1 mmol) $Co(NO_3)_2$·6H2O, 40.4 mg (0.1 mmol) $Fe(NO_3)_3$·6H2O, 24.2 mg (0.1 mmol) $Cu(NO_3)_2·3H_2O$, and 27.32 mg (0.1 mmol) $MoCl_5$, were dissolved in 10 ml of ethanol and sonicated for 10 min, as solution II. Under stirring, the solution II was quickly added to the solution I and continue stirring for 12 h. The black powder was obtained by drying with a rotary evaporator. The obtained black powder was loaded into a graphite tube which is sealed for further Joule-heating treatment. The NiCoFeCuMo (HEA-Mo) sample was produced by applying a voltage of 40 V and a current of 500 A for 2-3 seconds. Under the same conditions, $MoCl_5$ was replaced by 29.7 mg (0.1 mmol) $Zn(NO_3)_2·6H_2O$, or 25.1 mg (0.1 mmol) $Mn(NO_3)_2·4H_2O$, to obtain NiCoFeCuZn(HEA-Zn), or NiCoFeCuMn (HEA-Mn),

respectively.

PP separator modification

In a typical procedure, a mixture containing 90% HEA-X, and 10% PVDF was dissolved in N-methyl-2-pyrrolidone (NMP). The slurry was then coated onto a Celgard 2500 PP separator, followed by vacuum drying at 45 °C overnight to obtain the HEA-X@PP separators.

Fabrication of S Cathodes

Typically, S powder and Ketjen Black (8:2 in weight) were mixed and sealed in a Teflon contain filled with argon, followed by heating to 155 °C in an oven for 12 h. In the standard procedure, a mixture containing 80% S/KB, 10% Super P, and 10% PVDF was dissolved in NMP, coated on aluminum foil, and dried in vacuum at 60 °C for 24 h. The electrode is cut into 12 mm disks, and the sulfur load of each disk is 1.0 mg cm^{-2}.

Electrochemical measurements

A typical CR2032 coin cell lithium-sulfur battery is assembled with an S/KB cathode, a lithium foil anode, and a Celgard 2500 separator or HEA-X modified separator. For room temperature sodium-sulfur batteries, the cathode, the glass fiber separator (Whatman GF/D), HEA-Mo modified separator, and sodium metal anode were stacked in a CR2032 coin cell with 1.0 M $NaPF_6$ in DOL/Diglyme (v/v, 1:1) as the electrolyte. The electrolyte contained LiTFSI (1.0 M) and $LiNO_3$ (0.1 M) in a solvent mixture of DOL/DME (v/v, 1:1). The electrolyte/S rate was controlled at 20 μL mg^{-1} for routine sulfur mass loadings. The Li-S cells were cycled at a voltage window of 1.7-2.7 V, and the Na-S cells were cycled at a voltage range of 0.5-2.8 V. Cyclic voltammetric (CV) measurements were under a VMP3 multichannel electrochemical workstation (BioLogic, France) and electrochemical impedance spectroscopy (EIS) was conducted with a voltage amplitude of 10 mV in the frequency range 100 kHz to 10 mHz.

Materials Characterization

X-ray diffraction (XRD, Ultima IV Rigaku) analysis using Cu Kα radiation (λ = 1.5405 Å) at a voltage of 40 kV and a current of 40 mA was carried out. Scans were conducted with a step size of 0.02° in the 2θ range of 35-80°. As for scanning electron microscopy (SEM), an accelerating voltage of 10 kV was utilized, and the samples were analyzed

using a JSM-7900F instrument. Elemental analysis was performed in conjunction with energy-dispersive X-ray spectroscopy (EDS). Tunneling electron microscopy (TEM) was conducted using a Talos F200S instrument at an acceleration voltage of 200 kV. The chemical composition of the products was investigated using X-ray photoelectron spectroscopy (XPS) with a Thermo Scientific K-Alpha$^+$ instrument. The UV-vis spectra were obtained using a UV-2700 spectrophotometer from Shimadzu. The in-situ Raman spectra were recorded using a LabRAM HR Evo spectrometer from HORIBA FRANCE SAS at room temperature, with an excitation wavelength of 532 nm. Joule heating device purchased from Hefei In-situ Technology Co., Ltd.

Visual Encapsulation of LiPSs

The Li_2S_6 solution was prepared by stirring Li_2S and sulfur at a molar ratio of 1:5 overnight in a mixture of DOL and DME (v/v = 1:1). A Na_2S_6 solution was prepared by dissolving S and Na_2S in a mixture of DOL and Diglyme (v/v=1:1) in the ratio of 5:1 and stirring at 80 °C overnight. The experiments were conducted within an argon-filled glove box. An H-shaped glass cell containing tubes on two sides was used for the Li_2S_6 solution permeation tests. Separators (with/without HEA-X) divided the two tubes. A certain amount of Li_2S_6 solution was placed on the left side, and an equal volume of DOL/DME solvent was placed on the other side. Simultaneous photographs were taken during different time periods to record infiltration.

Preparation of symmetric cells

Two identical carbon paper (CP) electrodes were assembled, and 30.0 μL Li_2S_6 catholyte (0.2 M Li_2S_6 in DOL/DME) was added. The CV test of symmetric cells was tested on the electrochemical workstation. CV curves were obtained at a scan rate of 2 mV s^{-1} with a voltage range of -1.0 to 1.0 V.

Lithium polysulfide adsorption tests

The as-prepared Li_2S_6 solution was diluted to 50 mM by DME, and 50 mg catalysts (HEA-X) were added to immerse for 12 h.

Li_2S Precipitation Measurement

The electrode material and lithium foil are used to assemble the battery, and 25 μL blank electrolyte (1 M LiTFSI in tetreglyme) is dripped on one side of the lithium foil

and 25 μL Li_2S_8 catholyte (0.2 M Li_2S_8, 1 M LiTFSI in tetreglyme) is dripped on one side of the electrode material. The Li-S batteries were galvanostatically discharged at 0.112 mA to 2.06 V, and then potentiostatically discharged at 2.05 V for 36000 s.

Assembly of Li || Li symmetric cells

All Li-Li symmetric cells were assembled in an Argon-filled glove box (< 1 ppm of O_2 and H_2O). The electrolyte was consistent with that used in conventional Li-S batteries, and HEA-X double-sided modified separators and pristine PP separators were used, respectively.

Density functional theory (DFT) calculations

The quasicrystalline structure of the high-entropy alloy (HEA) was generated using the MCSQS (Monte Carlo Simulated Quenching) method[1] implemented in the Alloy Theoretic Automated Toolkit (ATAT) package.[2] The atomic arrangement in the high-entropy alloy was refined through stochastic shuffling of atoms, with the acceptance probability for each Monte Carlo step governed by the Metropolis-Hastings algorithm.[3] Following the Monte Carlo optimization of HEA structures, density functional theory (DFT) calculations were conducted using the Vienna ab initio Simulation Package (VASP)[4] to predict the material's catalytic properties. The Perdew-Burke-Ernzerh of (PBE) exchange-correlation functional within the generalized gradient approximation (GGA)[5] was adopted to describe the exchange-correlation interaction. A plane-wave cutoff energy of 450 eV was employed to ensure convergence. A vacuum layer of 15 Å was inserted along the z-axis to eliminate periodic image interactions. The van der Waals (vdW) interactions were corrected via the DFT-D3 correction scheme.[6]

The adsorption energy was calculated using the formula:

$$E_{ads} = E_{total} - E_{catalyst} - E_{adsorbate}$$

Here, E_{total}, $E_{catalyst}$, and $E_{adsorbate}$ denote the total energy of the adsorbed system, the energy of the clean catalyst, and the energy of the isolated adsorbate, respectively.

The Gibbs free energy (ΔG) for each step was computed as follows:

$$\Delta G = \Delta E + \Delta ZPE - T\Delta S$$

Here, ΔE, ΔZPE, and $T\Delta S$ represent the energy difference, zero-point energy (ZPE) difference, and temperature-entropy product between products and reactants,

respectively. The ZPE and entropy corrections were computed using the VASPKIT post-processing tool.[7]

Supporting Figures

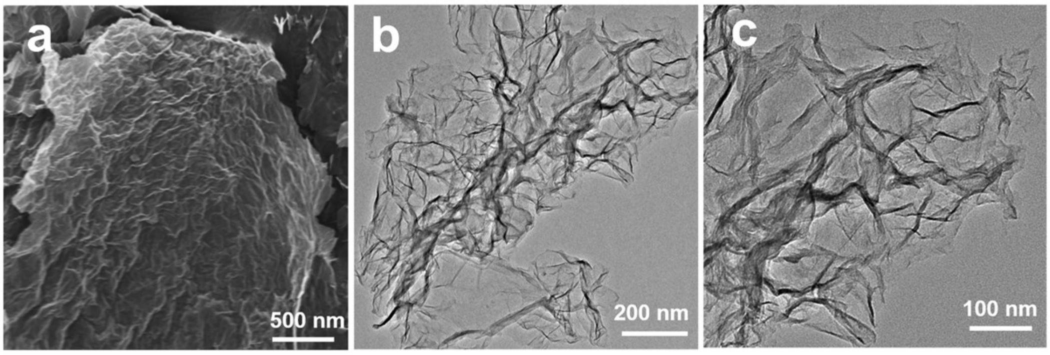

Figure S1. SEM (a) and TEM (b and c) images of rGO.

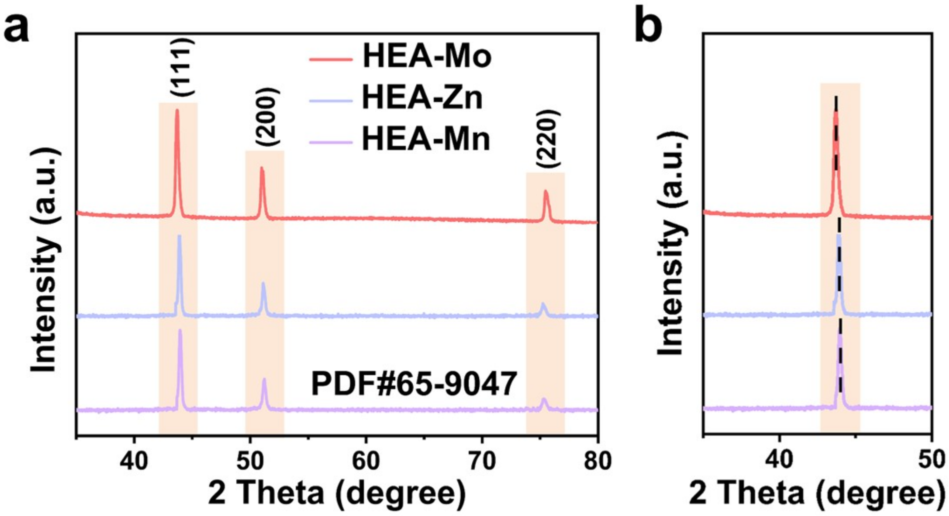

Figure S2. (a) XRD patterns of HEA-X. (b) Localized enlargement of the (111) peak in Figure (a).

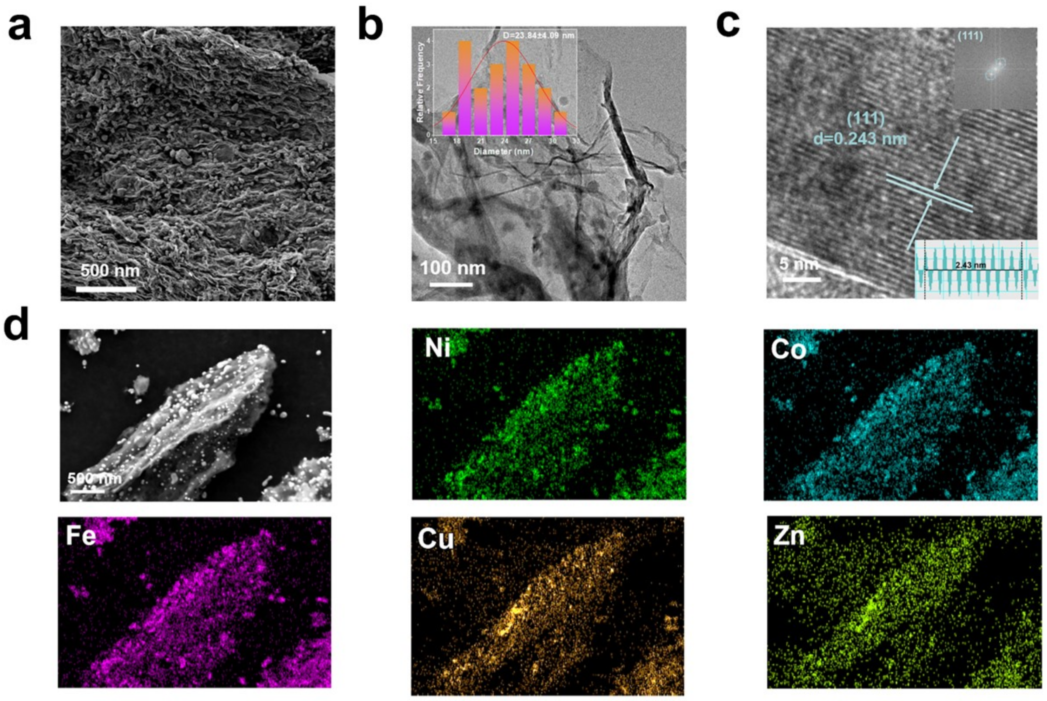

Figure S3. Characterizations of HEA-Zn: (a) SEM images, (b) TEM images, (c) HRTEM image with corresponding IFFT patterns, and (d) EDS elemental distribution in the SEM system.

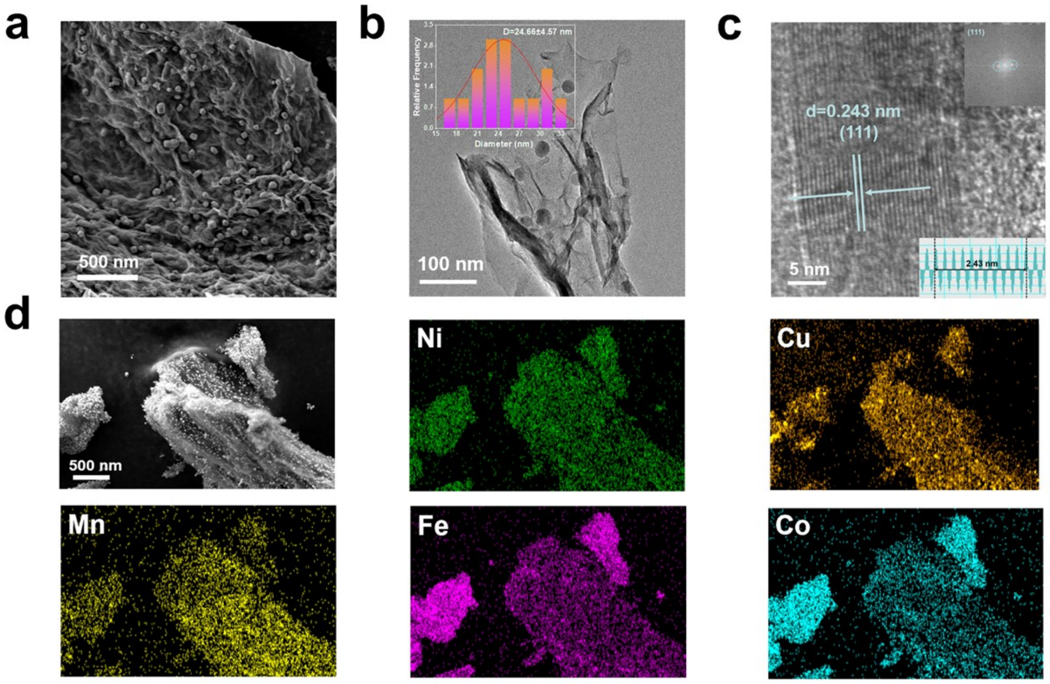

Figure S4. Characterizations of HEA-Mn: (a) SEM images, (b) TEM images, (c) HRTEM image with corresponding IFFT pattern, and (d) EDS elemental distribution in the SEM system.

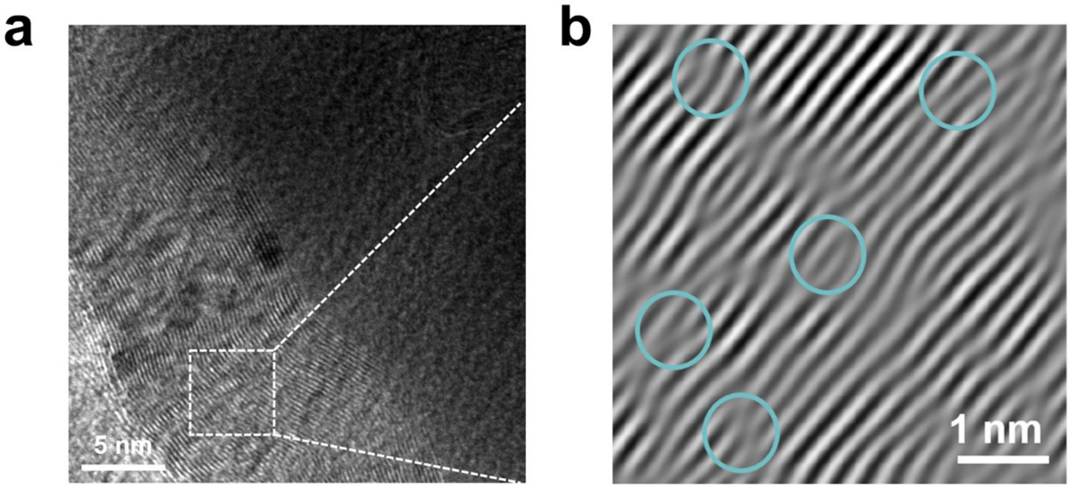

Figure S5. (a) HRTEM image of HEAs. (b) IFFT patterns of the boxed section in the left figure.

Alloying multiple elements of different sizes into a common lattice can lead to lattice distortions that cause the constituent atoms to deviate from their ideal positions, resulting in lattice distortion effects. The obvious lattice distortion phenomenon can be seen in Figure (b). It should be noted that lattice distortions are observed from three different high-entropy alloy (HEA-X) nanoparticles. The lattice distortion of high-entropy alloys significantly enhances the catalytic activity by modulating the electronic structure, optimizing the adsorption energy, and stabilizing the defect sites.

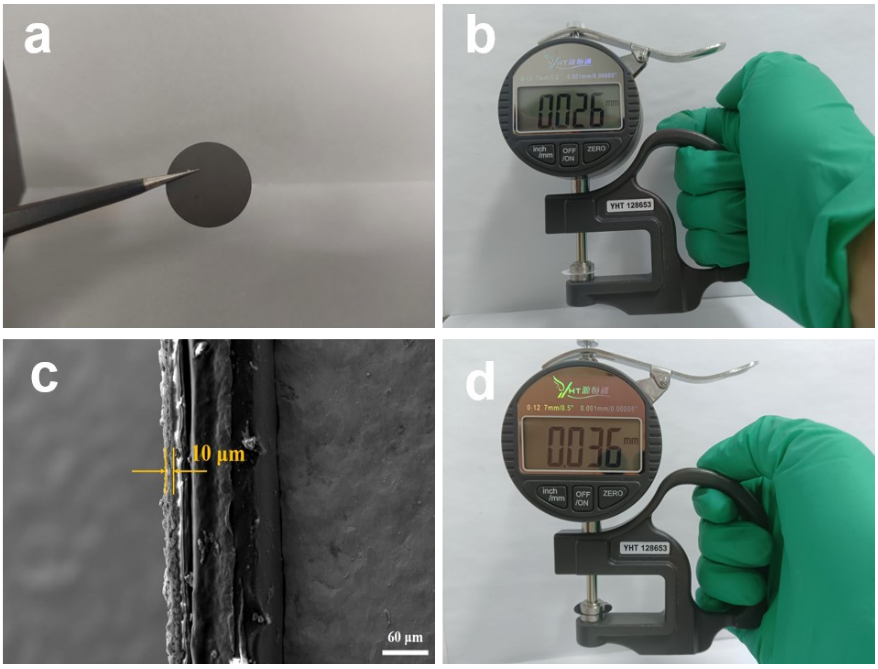

Figure S6. (a) Schematic diagram of the modified separators, (c) Cross-section of the separator taken by SEM, thickness of the pristine PP (b) and (d) modified separator measured by a thickness gauge.

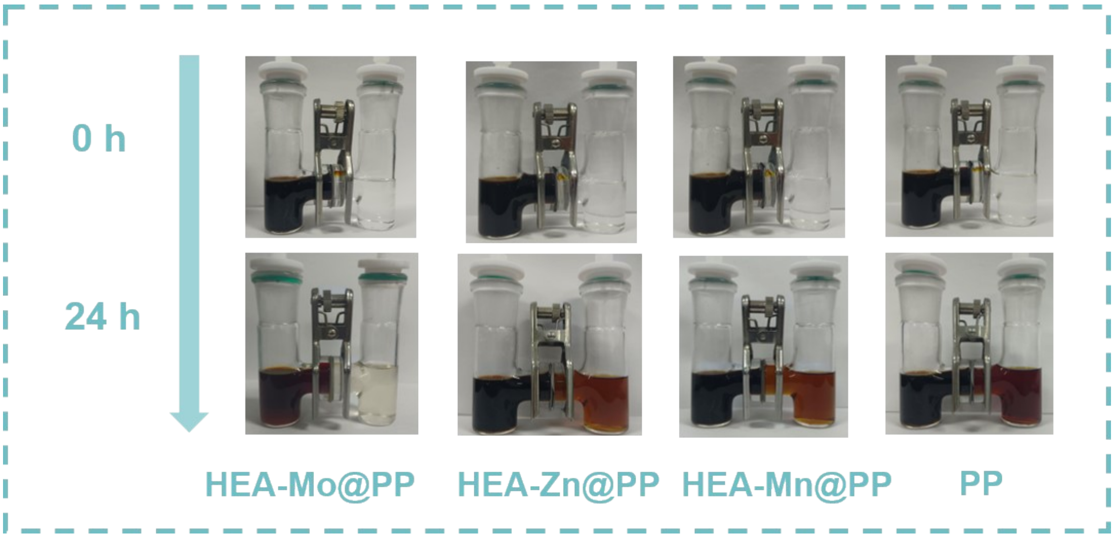

Figure S7. Visual inspection of polysulfide diffusion through HEA-Mo@PP, HEA-Zn@PP, HEA-Mn@PP and PP separators, respectively.

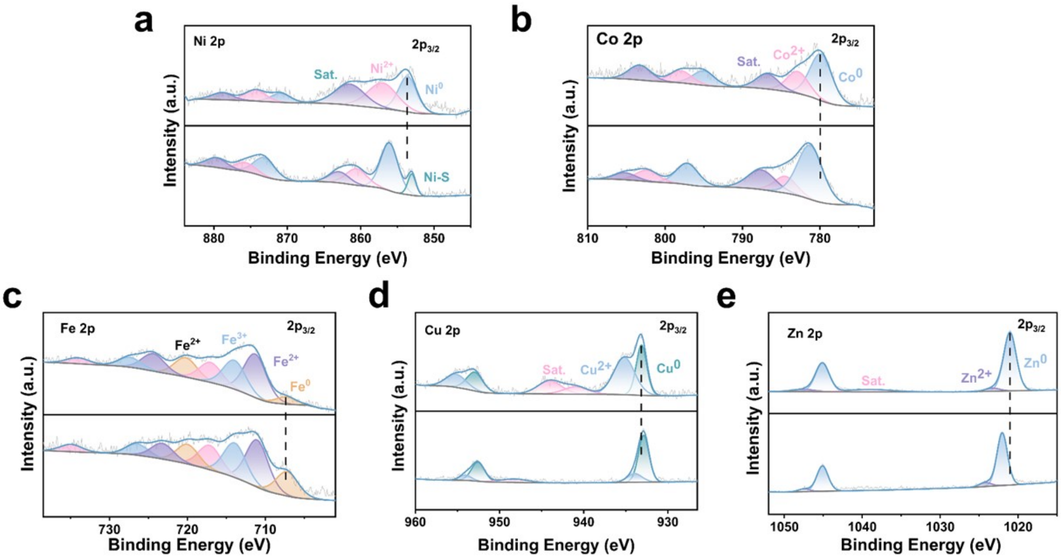

Figure S8. (a-e) High resolution XPS of Ni 2p, Co 2p, Fe 2p, Cu 2p and Zn 2p of HEA-Zn before and after adsorption of Li_2S_6.

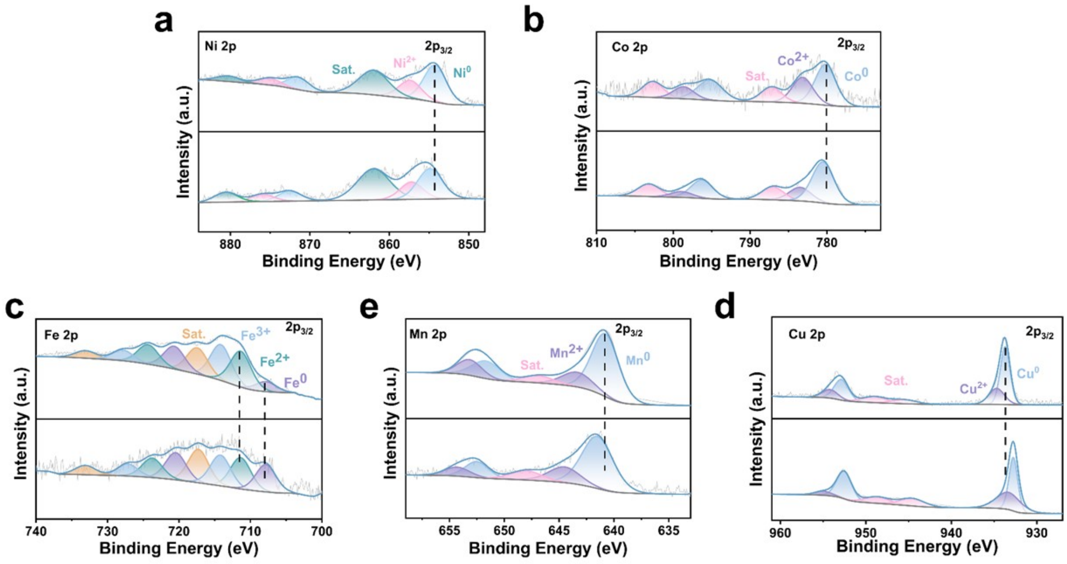

Figure S9. (a-e) High resolution XPS of Ni 2p, Co 2p, Fe 2p, Mn 2p, and Cu 2p of HEA-Mn before and after adsorption of Li_2S_6.

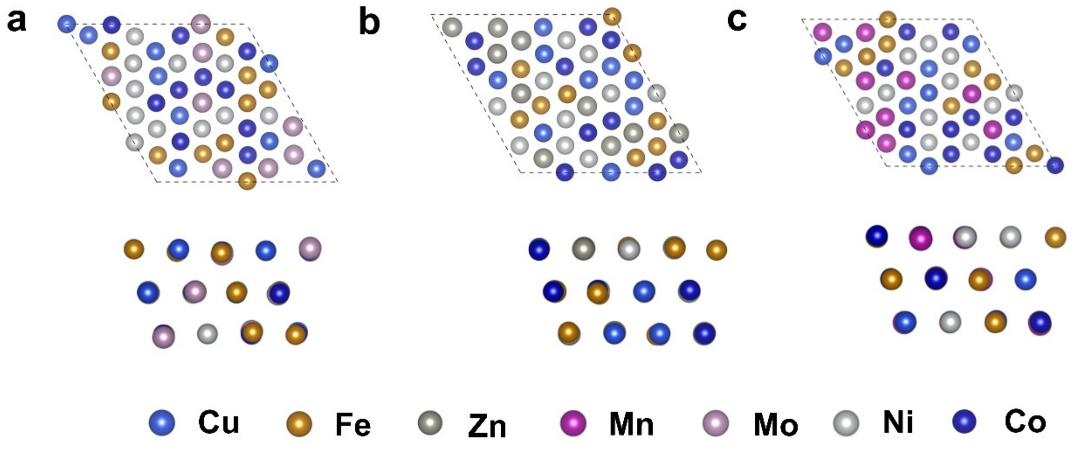

Figure S10. Initial configurations of HEA-Mo (a), HEA-Zn (b), HEA-Mn (c).

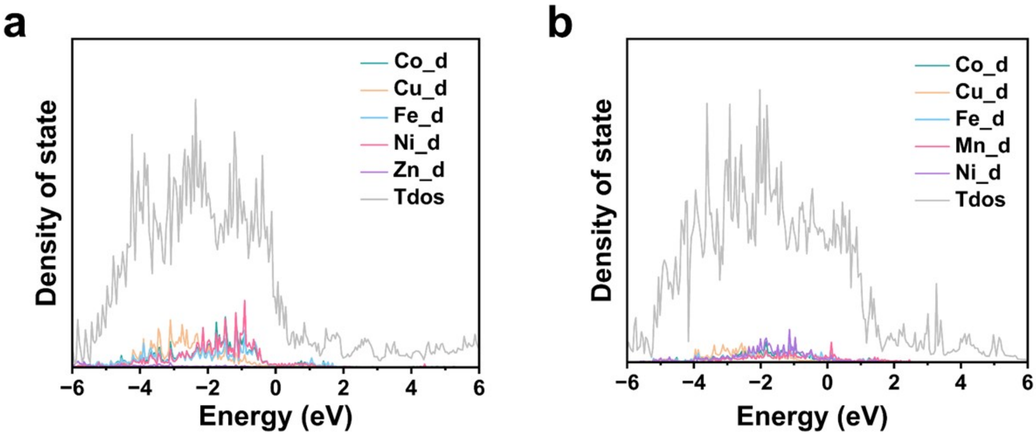

Figure S11. Density of States (DOS) of HEA-Zn (a) and HEA-Mn (b).

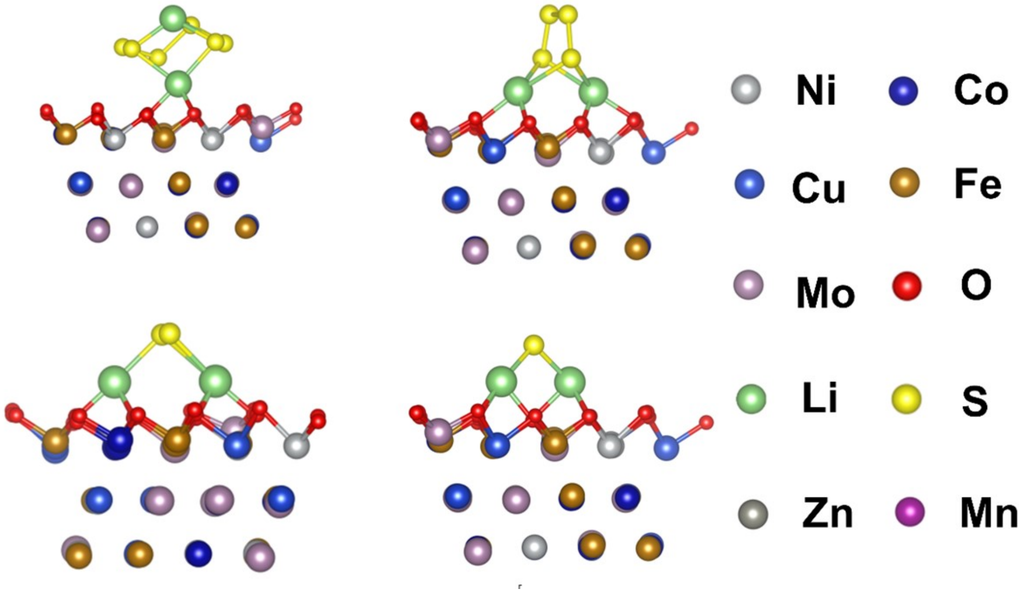

Figure S12. Optimized adsorption geometries of sulfur species adsorbed on the (111) surface of HEA-Mo.

Note: The different atoms in the above diagram are represented by different colored spheres. The Zn and Mn atoms have been labeled here and will not be repeated later.

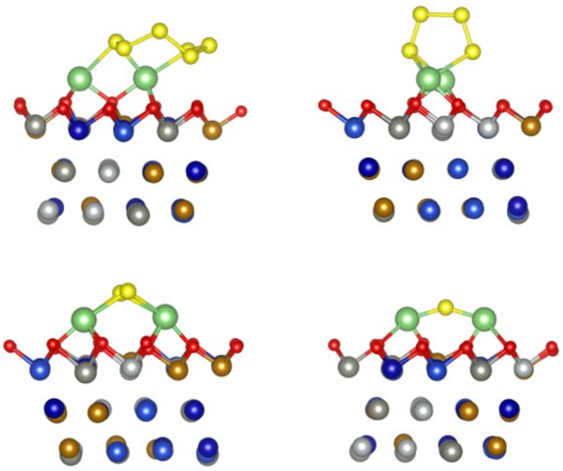

Figure S13. Optimized adsorption geometries of sulfur species adsorbed on the (111) surface of HEA-Zn.

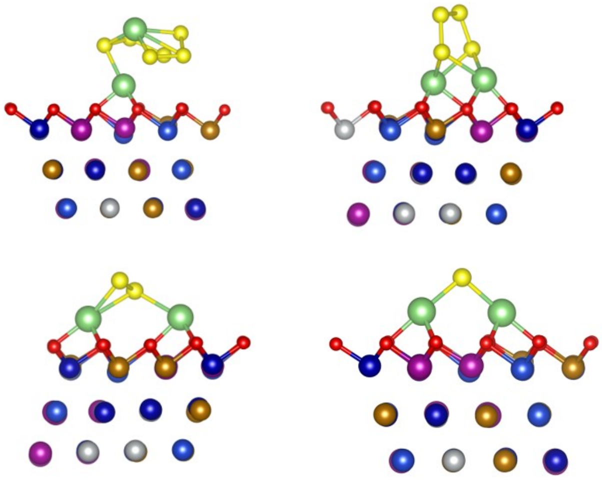

Figure S14. Optimized adsorption geometries of sulfur species adsorbed on the (111) surface of HEA-Mn.

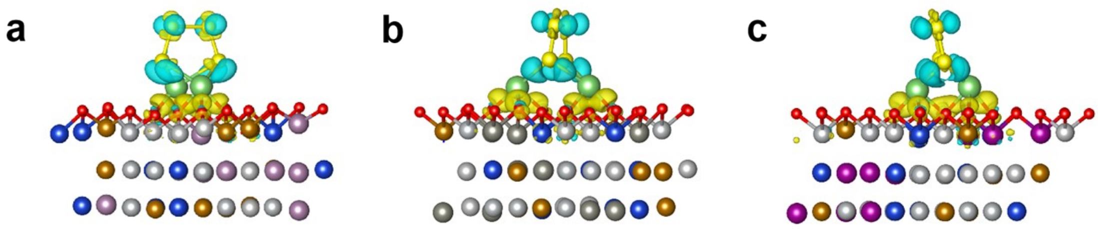

Figure S15. Differential charge density diagrams of Li_2S_4 on the (111) surface of HEA-Mo (a), HEA-Zn (b), and HEA-Mn (c). The isosurface level is set as 0.005 e/$Å^3$.

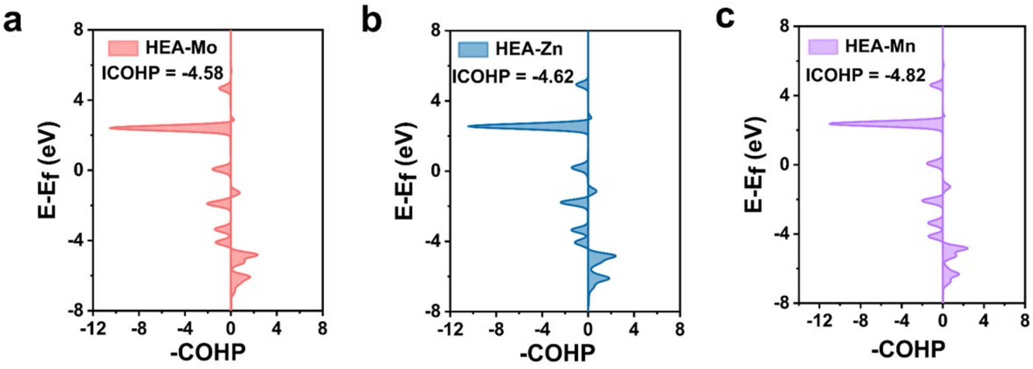

Figure S16. The crystal orbital Hamilton population (COHP) of S-S bond in Li_2S_4 absorbed on (111) plane of HEA-Mo (a), HEA-Zn (b), and HEA-Mn (c).

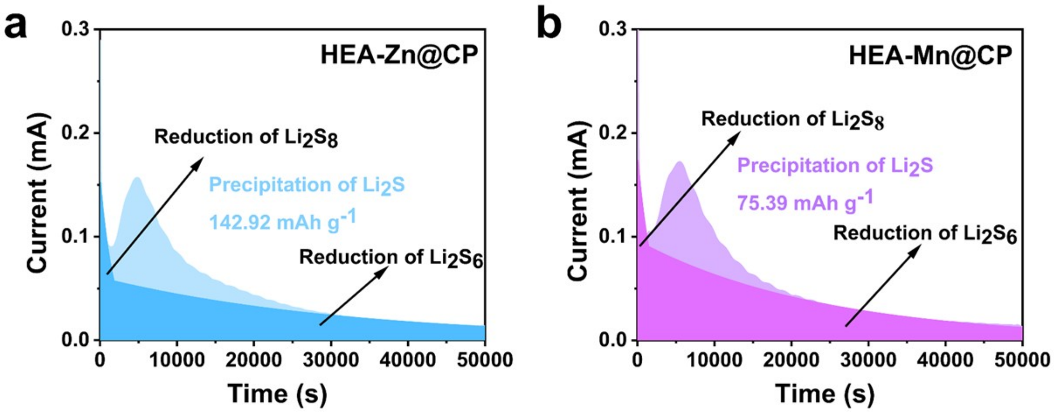

Figure S17. Li_2S deposition measurements for HEA-Zn (a) and HEA-Mn (b), with potentiostatic discharge profile of 2.05 V.

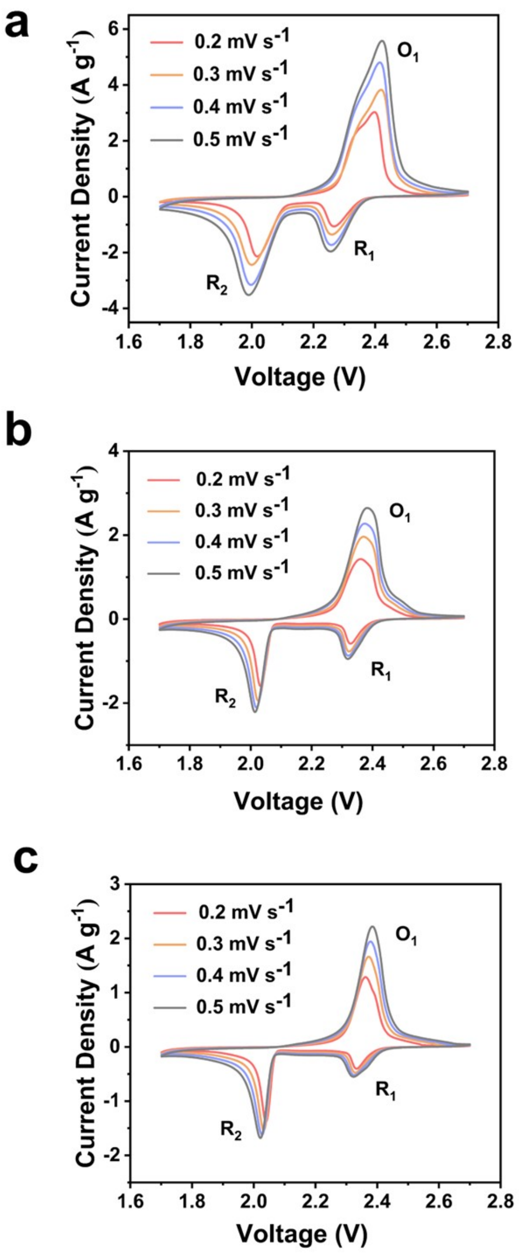

Figure S18. CV curves for (a) HEA-Mo, (b) HEA-Zn, and (c) HEA-Mn at various scan rates (0.2-0.5 mV s^{-1}).

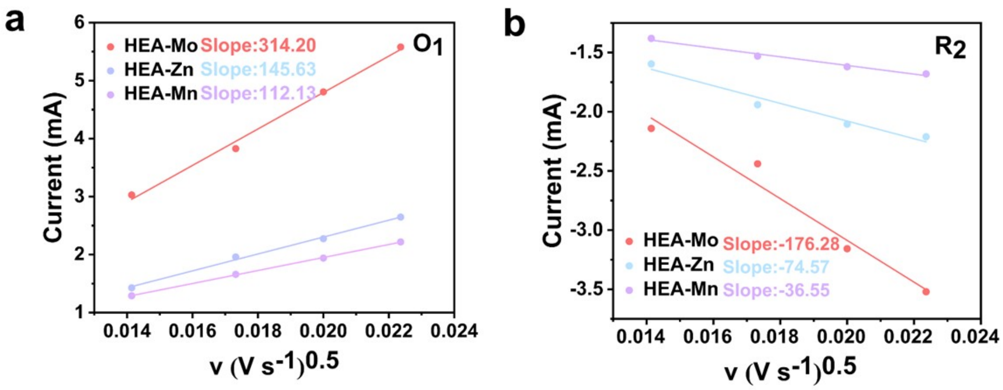

Figure S19. Calculated lithium ions diffusion coefficients of HEA-X. The lithium-ion diffusion coefficient D $_{Li+}$ (cm^2 s^{-1}) was measured by the CV test under different scan rates and calculated according to the Randles-Sevick equation:

$$I_p = 2.69 \times 10^5 \cdot n^{3/2} \cdot A \cdot D_{Li^+}^{1/2} \cdot c_{Li^+} \cdot v^{1/2}$$

where I_p stands for the peak current, A; n is the number of electrons transfer, which is 2 for Li-S batteries; A is the electrode area, cm^2; D $_{Li+}$ is the lithium-ion diffusion coefficient, cm^2 s^{-1}; C_{Li+} is the concentration of Li^+ in the electrolyte, mol mL^{-1}; v is the scan rate, V s^{-1}.

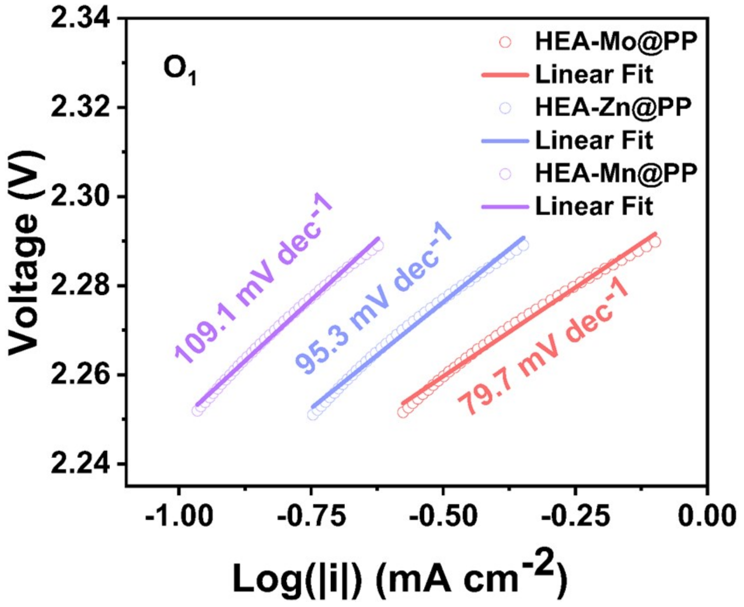

Figure S20. Tafel plots derived from CV curves at 0.2 mV s^{-1} calculated from the oxidation peaks O_1.

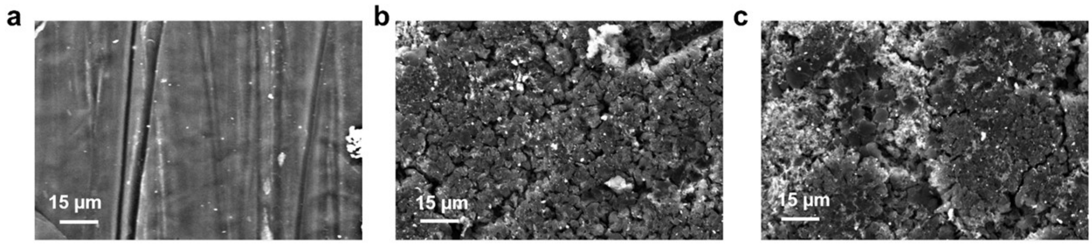

Figure S21. SEM images of Li-metal electrodes with (a) HEA-Mo@PP, (b) HEA-Zn@PP and (c) HEA-Mn@PP separators after cycling.

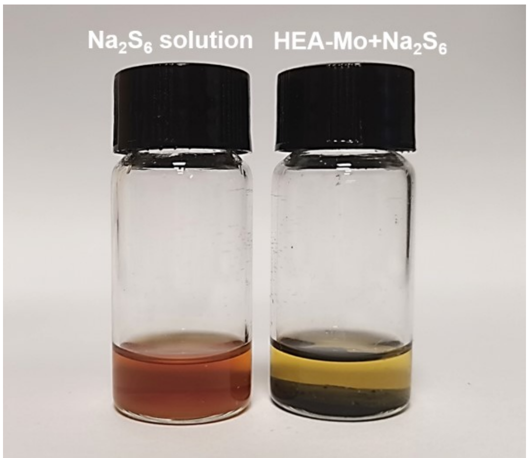

Figure S22. Photographs of pristine Na_2S_6 and Na_2S_6 solutions after being absorbed by HEA-Mo for 24 h.

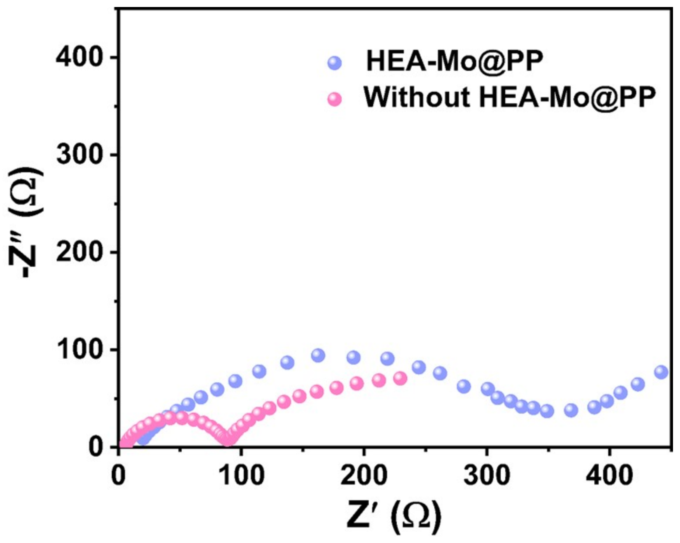

Figure S23. Nyquist plots for EIS profiles of room-temperature Na-S batteries with different separators.

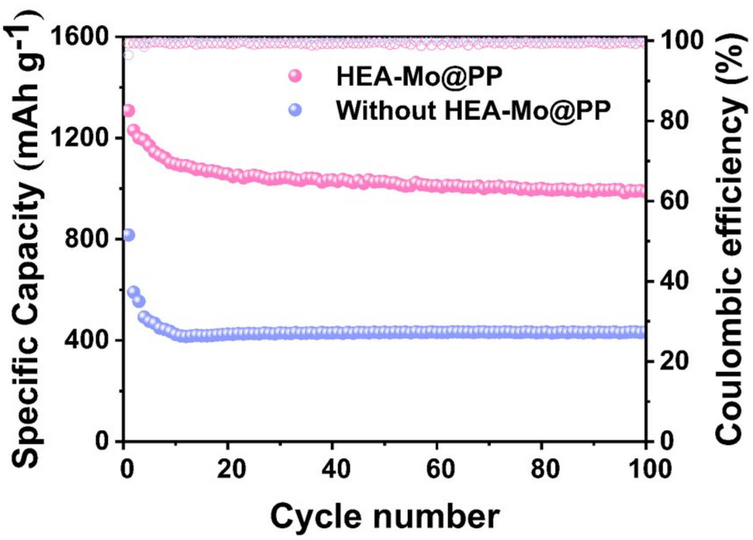

Figure S24. Cycling performance of different separators at 0.2 C.

Table S1. Atomic ratio of HEA-X and their ΔS_{mix}

	Ni	Co	Fe	Cu	X	S_{mix}
HEA-Mo	0.25	0.25	0.3	0.1	0.1	1.515R
HEA-Zn	0.2	0.2	0.3	0.25	0.05	1.501R
HEA-Mn	0.2	0.2	0.3	0.2	0.1	1.557R

The configurational entropy of materials was calculated by the following equation:

$$\Delta S_{mix} = -R(\sum_{i=1}^{n} xilnxi)$$

where R is the molar gas constant and x_i is the molar content of the i_{th} metal species(n≥i≥1).

Table S2. Comparation of reported Li-S batteries with different electrocatalysts

Category	Catalyst	Rate	Cycle	Decay	Ref
**Metal-based catalyst**	Transition metal nanoparticles	2 C	500	0.064%	Ref.[8]
	Metal phosphides	1 C	500	0.038%	Ref.[9]
	Metal sulfides	1 C	600	0.06%	Ref.[10]
	Metal oxides	1 C	500	0.033%	Ref.[11]
**Carbon-based catalyst**	Porous carbon materials	2 C	1000	0.039%	Ref.[12]
	Doped carbon materials	1 C	500	0.055%	Ref.[13]
	Composite carbon materials	1 C	800	0.047%	Ref.[14]
**Metal-organic frameworks (MOFs)**	HPC-MOF-M	1 C	500	0.053%	Ref.[15]
	FCMMX	0.5C	800	0.056%	Ref.[16]
**High Entropy Alloys (HEAs)**	**HEA-Mo@PP**	**1 C**	**2000**	**0.027%**	**This work**

References

1 X. W. Zhou, H. N. G. Wadley, R. A. Johnson, D. J. Larson, N. Tabat, A. Cerezo, A. K. PetfordLong, G. D. W. Smith, P. H. Clifton, R. L. Martens and T. F. Kelly, *Acta Mater.*, 2001, **49**, 4005–4015.

2 A. Van De Walle, M. Asta and G. Ceder, *Calphad*, 2002, **26**, 539–553.

3 B. Calderhead, *Proc. Natl. Acad. Sci.*, 2014, **111**, 17408–17413.

4 G. Kresse and J. Furthmüller, *Phys. Rev. B*, 1996, **54**, 11169–11186.

5 J. P. Perdew, J. A. Chevary, S. H. Vosko, K. A. Jackson, M. R. Pederson, D. J. Singh and C. Fiolhais, *Phys. Rev. B*, 1992, **46**, 6671–6687.

6 S. Grimme, J. Antony, S. Ehrlich and H. Krieg, *J. Chem. Phys.*, 2010, **132**, 154104.

7 V. Wang, N. Xu, J. Liu, G. Tang and W. Geng, *Comput. Phys. Commun.*, 2021, **267**, 108033.

8 C. Yang, F. Wang, D. You, W. Yang, Y. Wang, W. Han, Y. Zhang, Z. Zhu and X. Li, *Chem. Eng. J.*, 2024, **496**, 153812.

9 B. Li, P. Wang, J. Yuan, N. Song, J. Feng, S. Xiong and B. Xi, *Angew. Chem. Int. Ed.*, 2024, **63**, e202408906.

10 X. Wang, S. Liu, J. Yang, S. He and J. Qiu, *Adv. Energy Mater.*, 2024, **14**, 2400104.

11 H. Wang, C. Xu, X. Du, G. Liu, W. Han and J. Li, *Chem. Eng. J.*, 2023, **471**, 144338.

12 G. Li, J. Sun, W. Hou, S. Jiang, Y. Huang and J. Geng, *Nat. Commun.*, 2016, **7**, 10601.

13 J. Pan, Y. Sun, Y. Wu, J. Li, W. Huang, K. Shi, Y. Lin, H. Dong and Q. Liu, *Carbon*, 2022, **198**, 80–90.

14 X. Zuo, M. Zhen, D. Liu, L. Fu, Y. Qiu, H. Liu and Y. Zhang, *Adv. Funct. Mater.*, 2024, **34**, 2405486.

15 L. Xie, Y. Xiao, Q. Zeng, Y. Wang, J. Weng, H. Lu, J. Rong, J. Yang, C. Zheng, Q. Zhang and S. Huang, *ACS Nano*, 2024, **18**, 12820–12829.

16 P. Yang, J. Qiang, J. Chen, Z. Zhang, M. Xu and L. Fei, *Angew. Chem. Int. Ed.*, 2025, **64**, e202414770.
